# Curcumin, Cardiometabolic Health and Dementia

**DOI:** 10.3390/ijerph15102093

**Published:** 2018-09-24

**Authors:** Yoona Kim, Peter Clifton

**Affiliations:** 1 Department of Food and Nutrition/Institute of Agriculture and Life Science, Gyeongsang National University, Jinju 52828, Korea; 2 School of Pharmacy and Medical Sciences, University of South Australia, General Post Office Box 2471, Adelaide, SA 5001, Australia; peter.clifton@unisa.edu.au

**Keywords:** Curcumin, glucose, insulin resistance, inflammation, type 2 diabetes

## Abstract

Current research indicates curcumin [diferuloylmethane; a polyphenolic compound isolated from the rhizomes of the dietary spice turmeric (*Curcuma longa*)] exerts a beneficial effect on health which may be partly attributable to its anti-oxidative and anti-inflammatory properties. The aim of this review is to examine potential mechanisms of the actions of curcumin in both animal and human studies. Curcumin modulates relevant molecular target pathways to improve glucose and lipid metabolism, suppress inflammation, stimulate antioxidant enzymes, facilitate insulin signalling and reduce gut permeability. Curcumin also inhibits Aβ and tau accumulation in animal models and enhances mitochondria and synaptic function. In conclusion, in high-dose animal studies and in vitro, curcumin exerts a potential beneficial effect on cardiometabolic disease. However, human studies are relatively unconvincing. More intervention studies should be conducted with the new curcumin formulation with improved oral bioavailability.

## 1. Introduction

Type 2 diabetes Mellitus (T2DM) is associated with impaired insulin signalling, leading to hyperglycaemia and micro and macrovascular diseases [1,2]. Insulin resistance is a major contributor to the pathogenesis of T2DM with secondary pancreatic failure [2,3]. The prevalence of diabetes mellitus will increase worldwide from 451 million people aged over 18 in 2017 to 693 million people in 2045 [4]. Diabetes is an enormous social, financial and health system burden across the world [4,5]. Lifestyle modification, including a healthy diet, can lower the risk of T2DM [6]. Dietary polyphenols have been a major research focus to reduce the risk of T2DM [7,8,9,10]. This review aims to present an update on the effect of curcumin (a polyphenol) on the prevention and treatment of T2DM and cardiovascular disease (CVD) in animal studies and human studies.

## 2. Curcumin

**Figure 1:**
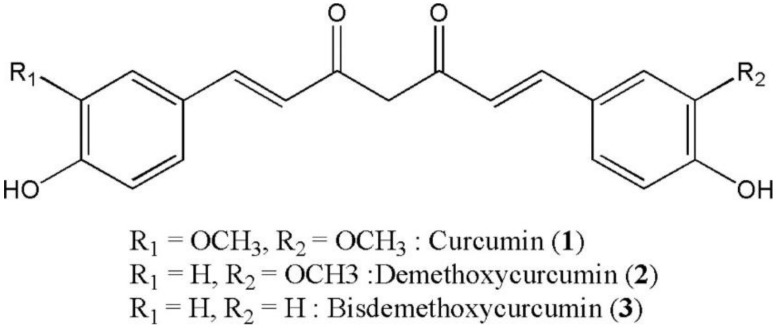
Chemical structure of curcuminoids.

Turmeric obtained from the dried *Curcumin longa* L. (ginger family) is a curry spice. This is widely consumed as a food ingredient and has long been used for medicinal purposes in China and Southeast Asia [11,12,13]. Turmeric is comprised of 3 curcuminoids (curcumin, demethoxycurcumin and bisdemethoxycurcumin), sugars, proteins, volatile oils (natlantone, tumerone and zingiberone) and resins [14]. Of the 3 curcuminoids, curcumin is the most active lipophilic polyphenol compound which is quite stable in the acidic pH of stomach [15,16]. Curcumin is used as a food colorant (yellow), flavouring, and additive [13]. The chemical structure of pure curcumin (diferuloylmethane) is 1,7-bis-(4-hydroxy-3-methoxyphenyl)-1, 6-heptadiene-3,5-dione and comprises two ferulic acid residues joined by a methylene bridge [17]. The structure of curcumin is shown in Figure 1. Commercially available curcumin contains 77% curcumin, 17% demethoxycurcumin and 3% bisdemethoxycurcumin [13].

### 2.1. Curcumin Safety

The Allowable Daily Intake (ADI) value of curcumin is 3 mg/kg body weight [18]. Healthy subjects consumed curcumins ranging from 0.5 to 12 g/day for 72 h in order to assess the safety of curcumin. Up to 12 g/day of curcumin consumption for 72 h was deemed safe. About 30% subjects showed diarrhoea and headache, which were not dose-related [19]. Subjects who consumed curcumins at a dose ranging from 0.45 to 3.6 g/day for 1–4 months experienced nausea and diarrhoea. The serum concentrations of alkaline phosphatase and lactate dehydrogenase were elevated in 3–4 out of 15 patients [20].

A review article investigating the pharmacokinetic interactions of curcumin with conventional drugs (including cardiovascular drugs, antidepressants, anticoagulants, antibiotics, chemotherapeutic agents, and antihistamines) showed that curcumin can alter maximum serum concentrations (Cmax) and area under the curve (AUC) when used with those drugs. Curcumin can inhibit cytochrome P450 monooxygenases (drug-metabolising enzymes) and P-glycoprotein (an efflux pump from the ATP-binding cassette (ABC) super family which pumps various xenobiotics (e.g., drugs) out of cells. Only one clinical trial has demonstrated a significant interaction between curcumin and drugs [21].

### 2.2. Curcumin Bioavailability

Detectable concentrations of curcumin and its metabolites in both blood and urine were observed with curcumin ingestion of ≥3.6 g/day in several studies [20,22,23,24,25]. The mean plasma concentration in patients with advanced colorectal cancer refractory to standard chemotherapies who consumed 3.6 g/day of curcumin for up to 4 months was 4.3 ng/mL (i.e., about 0.01 μM/L). The mean plasma concentrations of curcumin glucuronide and curcumin sulphate were 5.8 and 3.3 ng/mL, respectively, 1 h after administration [20]. The plasma concentrations (mean ± SD) of curcumin for patients with high-risk or pre-malignant lesions who took 4 g, 6 g and 8 g daily for 3 months were 0.19, 0.20, and 0.60 µg/mL, respectively [22]. Healthy subjects (*n* = 6) ingested 10 g of curcumin extract after hydrolysis of conjugates. The Cmax (mean ± SE) of curcumin conjugates detected as glucuronide and sulphate were 3.2 ± 0.56 µg/mL. These values were 1000 times higher than those of other study subjects with lower doses of curcumin (mentioned above [20,22]). The time to reach maximum plasma concentrations (Tmax) was 4.33 ± 3.2 h [25].

However, other studies [19,22,24,25,26,27,28,29] showed very low or even undetectable concentrations of blood curcumin after oral consumption. This may be attributable to chemical instability, low absorption, rapid metabolism, and enhanced elimination [30]. Animal studies showed that >90% of ingested curcumin is excreted in the faeces [31]. Microbial metabolites of curcumin have been reported [32,33,34,35,36].

Efforts to enhance curcumin bioavailability have been made, including systemic implants, curcumin nanoparticles or curcumin with stabilised surfactants [37,38,39,40,41,42].

Different formulations are available to increase curcumin solubility, circulation, permeability, bioavailability and resistance to metabolic processes using various materials, such as adjuvants (piperine), bio-conjugates [turmeric oil, glycine, alanine and epigallocatechin-3-gallate (EGCG)], lipids (phospholipid), nanoparticles (liposome, micelles, noisome, nanogels, chitosan, gold, silver, cyclodextrin, dendrimer, solid lipids), protein (BSA, soy protein isolated) and others (hyaluronic acid, hydrogel, polymer, PEG-PEI emulsion, polymer encapsulated, beta-lactoglobulin) [26,43,44]. Adjuvant piperine (extracted from black pepper, which suppresses rapid glucuronidation of curcumin in liver and intestine and then decreases urinary excretion of curcumin), when used with curcumin, increases free curcumin in tissues leading to increased curcumin bioavailability by ~2000 times [26]. Human interventions showed a favourable effect of the formulation of curcumin with piperine on anthropometric parameters and lipid profiles [45,46].

Curcumin bioavailability increased 7 to 8 times when curcumin was bio-conjugated with turmeric oil (Biocurcumax^TM^ Arjuna Natural Extracts Ltd., Kerala, India) [47]. The formulation of curcumin bio-conjugates with epigallocatechin-3-gallate (EGCG) [48] and with glycine, alanine and turmeric oil [49] enhanced curcumin uptake into cells in vitro. The phospholipid-curcumin complex enhanced bioavailability [50]. A liposome (25–205 nm in diameter)-curcumin complex [43] showed higher bioavailability than unformulated curcumin.

Noisomes (190–1140 nm in diameter) showed increased curcumin bioavailability [51]. A curcumin micelle (10–100 nm in diameter) with a nano-sized core and a membrane of hydrophilic polymer showed enhanced bioavailability [43,52,53,54,55,56]. In a human intervention, nano-micelle curcumin showed beneficial effects on glucose and lipid profiles in diabetic patients [57]. Moreover, nanogel (10–200 nm in diameter) [58], chitosan (100–250 nm in diameter) [59,60], gold (200–250 nm in diameter) [61,62], silver (~15 nm in diameter) [63], cyclodextrin [64], dendrimer (15–150 nm in diameter) [65] and solid lipids (50–1000 nm in diameter) [66] showed improved solubility and bioavailability of curcumin in vitro and animal studies.

A curcumin-solid lipid nanoparticle (SLNP), called “Longvida^®^” (Verdure Sciences, Noblesville, IN, USA) showed improved bioavailability with a plasma concentration of 0.1–0.2 µM [67]. In a human intervention, Longvida^®^ showed favourable effects on lipid profiles and memory and mood [68].

Modified curcumin chemical structures (analogues or derivatives of curcumin) have been developed with enhanced stability, solubility, bioavailability and biological effects. Rapid absorption (2 h and 57 min) and slow elimination (3 h and 39 min half-life) were shown [69]. Bioavailability was 60% when 32 mg/kg of curcumin analogue (EF-24) was orally administrated to mice [69].

The first curcumin nanoparticle (Theracurmin^®^, Theravalues Corp., Kioicho Chiyoda-ku, Tokyo, Japan) showed enhanced bioavailability in healthy subjects [70]. The t1/2 was 9.7 ± 2.1 h for 150 mg and 13.0 ± 3.3 h for 210 mg and plasma curcumin was still detectable at the 24-h time point [70]. In a human intervention, Theracurmin^®^ with 90 mg of curcumin twice daily showed favourable effects on memory and attention [71].

## 3. Effects of Curcumin on Cardiometabolic Health

A summary of curcumin human intervention studies is shown in Table 1.

### 3.1. Anti-Oxidative Effects

Oxidative stress is characterised by an imbalance between reactive oxygen species (ROS) generation and anti-oxidative defence [72]. Hyperglycemia promotes autooxidation of glucose, glycation of protein and enhanced polyol pathways leading to the increased ROS [73]. Continuous oxidative stress can cause chronic inflammation which may result in chronic diseases such as T2DM, CVD and Alzheimer’s disease (AD) [74,75,76,77].

The anti-oxidant effect of curcumin as a free radical scavenger appear to be related to its phenolic O-H and C-H groups [78].

#### 3.1.1. Human Studies

There are fewer than 30 human studies, and most are small and uncontrolled, so the data is relatively unconvincing. However, it is noted that volunteer characteristics and experimental designs were rarely the same in different studies.

Thirty-eight healthy middle-aged subjects (40–60 years old) who consumed a low dose of lipidated curcumin (80 mg/day) for 4 weeks showed some favourable effects on cardiometabolic health, but no comparison was made with placebo, so no firm conclusions can be drawn [79].

Yang et al. 2015 [80] conducted a small open uncontrolled study to see if curcumin intake in subjects with T2DM can lower urinary microalbuminuria excretion. Urinary microalbuminuria was significantly decreased by 70% (*n* = 14; *p* < 0.05) by 500 mg/day of curcumin for 15 days. Moreover, decreased levels of malondialdehyde (MDA) and increased levels of nuclear factor erythroid 2-related factor 2 (Nrf2), NAD(P)H: quinone oxidoreductase (NQO1), superoxide dismutase (SOD) were observed. The levels of lipopolysaccharides (LPS) and caspase 3 decreased. The levels of IκBα and gut barrier function increased in a before and after comparison. There were no significant differences in fasting blood glucose, insulin, C-peptide, triglyceride (TG), total cholesterol (TC), HDL-C, low-density lipoprotein (LDL-C), alanine transaminase (ALT), aspartate transaminase (AST), creatinine and urea nitrogen (BUN) following 15-day curcumin ingestion compared with the baseline [80].

In a randomised, double-blind, placebo-controlled, crossover study, 62 overweight/obese women aged over 40–75 years (mean body mass index (BMI) ≥ 34.5 ± 0.8 kg/m^2^) with C–reactive protein (CRP) = 8.05 ± 1.33 mg/L who were treated with 2.8 g/day of turmeric (~112 mg/day curcumin) for 4 weeks showed no changes in oxidative stress (F2-iso-prostanes, oxidised LDL-C), inflammatory markers (CRP, IL-6, IL-8, IL-10, tumour necrosis factor α (TNFα), IFNγ, IL-1β and IL-12p70), serum glucose, body weight, percent body fat, systolic blood pressure, augmentation index when compared with either the placebo or the baseline [81].

#### 3.1.2. Several Possible Mechanisms of Curcumin Anti-Oxidant Activity Have Been Proposed [82,83,84,85,86,87,88,89,90,91,92,93]

Severe oxidative stress can cause DNA damage and stimulate the DNA repair enzyme poly ADP-ribose polymerase-1 (PARP-1). Inhibition of PARP-1 reduces tissue injury in oxidative stress-related diseases (e.g., T2DM and CVD) [94,95]. Curcumin suppressed ROS in islets of streptozotocin-induced diabetic rats by increasing Cu/Zn SOD and reduced PARP-1 activity, which is a secondary response to inhibition of ROS-related damage [91].

Curcumin reduced thiobarbituric acid reactive substances (TBARS), lipid peroxidation and MDA, and increased antioxidant enzyme activities such as SOD, catalase (CAT), glutathione peroxidase (GPx) and glutathione-S-transferase (GST) in erythrocytes, liver, kidney, heart, pancreas and brain of diabetic animals [87,88,93] but very high doses were used (e.g., 100 mg/kg).

Curcumin (0–10 μm/L) reduced haemoglobin glycosylation and lipid peroxidation in erythrocytes under oxidative stress induced by high glucose concentrations [83], and inhibited aldose reductase which in turn leads to decreased intracellular sorbitol accumulation [84]. Elevated aldose reductase activities cause increased sorbitol production from glucose, which may increase diabetes complications [85,86]. It is noted that recent studies indicate aldose reductase inhibitors alone appear to be not effective for prevention of diabetic complications [96,97].

In alloxan-induced diabetic rats, a glucose-lowering of curcumin (1 g/kg body weight or 0.08 g/kg body weight) was noted, which led to reduced oxidative stress (decreased TBARS) through the prevention of glucose influx into the polyol pathway, as well as a decrease in sorbitol dehydrogenase (SDH—converts sorbitol to fructose) [82].

Curcumin (20 µm/L or 40 μm/L) activated the expression of antioxidant enzymes including γ-glutamyl-cysteine ligase (GCLM), NQO1 and heme oxygenase 1 (HO-1) in pancreatic β cells and adipocytes [89,98]. The expression of HO-1 was induced by curcumin through the activation of Nrf2/antioxidant-responsive element (ARE) pathway in renal epithelial cells [90]. It is noted that Nrf2 is closely associated with oxidative stress-induced inflammation [99].

### 3.2. Anti-Inflammatory Effects

A meta-analysis of 8 randomised controlled trials (RCTs) [79,100,101,102,103,104,105,106] in subjects with a variety of diseases showed that curcuminoids significantly lowered CRP levels (by a mean 2.2 mg/L) compared with a placebo [107]. In a meta-analysis of RCTs [108,109,110,111,112,113], curcumin significantly reduced TNF-α (weight mean difference −4.69 pg/mL; 95% CI: −7.10, −2.28; *p* < 0.001) [114]. In a meta-analysis of 9 RCTs [102,103,106,108,109,111,112,115,116] in subjects with different diseases, curcumin significantly lowered IL-6 by 0.6 pg/mL (*P* = 0.01) compared with control [117]. Curcumin supplement (1 g/day, *n* = 59) for 8 weeks significantly decreased TNF-α, IL-6, transforming growth factor beta (TGF-β) and monocyte chemoattractant protein 1 (MCP-1) compared with placebo [112]. In a randomised, double-blind, crossover trial of 30 obese subjects with BMI ≥ 30, curcumin treatment (1 g/day) for 4 weeks significantly decreased IL-4, IL-1 β and vascular endothelial growth factor (VEGF) without differences in IL-2, IL-6, IL-8, IL-10, IFN γ, epidermal growth factor (EGF), and MCP-1, compared with a placebo [111].

#### Curcumin and Inflammatory Pathways

A mitogen-activated protein kinase (MAPK) regulates embryogenesis, cell differentiation, proliferation, survival and death [118,119]. The MAPK pathway is comprised of a series of protein kinases [118,119,120]. The p38 MAPK pathway is associated with inflammatory responses [121,122].

TNF-α and LPS activate PI 3-kinase in human intestinal microvascular endothelial cells (HIMEC), and subsequently increase serine/threonine kinase (Akt) phosphorylation for Akt activation. Activated Akt stimulates MAPK and nuclear factor kappa B (NF-kB) signalling pathways resulting in vascular cell adhesion molecule (VCAM-1) and MAdCAM-1 expression in the intestine [123,124].

Curcumin suppressed nitric oxide, MCP-1, IL-1β, IL-6, TNF-α, cyclooxygenase-2 (COX-2) and prostaglandin E2 (PGE2) by suppressing Jun NH2-terminal kinase (JNK), extracellular signal-regulated kinase1/2 (ERK1/2) and p38MAPK in adipocytes in vitro [125,126,127] and in other organs [128,129,130,131].

Curcumin may reduce diabetes complications related to vascular inflammation. Hyperglycaemic conditions induce the secretion of proinflammatory cytokines via epigenetic changes, which are mediated by the opposing actions of histone deacetylases (HDACs) and histone acetylases (HATs) [132,133]. Curcumin treatment of human monocytic (THP-1) cells under high-glucose conditions increased HDAC2, decreased HATs activity and expressions of p300 and acetylated CBP/p300 gene expression, consequently leading to decreased NF-κB transcription activity and proinflammatory cytokine secretion (IL-6, TNF-α) [132].

Oxidative stress and inflammation are contributors to cardiometabolic disease including insulin resistance, T2DM, CVD and AD. The potent anti-oxidative and anti-inflammatory effects of curcumin could beneficially influence glucose and lipid homeostasis and endothelial function.

### 3.3. Glucose Homeostasis

In a randomised double-blind, placebo-controlled study, subjects with non-alcoholic fatty liver disease (NAFLD) (mean age 46.37 ± 11.57 years; mean BMI 31.35 ± 5.67 kg/m^2^; *n* = 77) consumed 500 mg/day of an amorphous dispersion curcumin formulation (equivalent to 70 mg curcumin) for 8 weeks. Curcumin consumption significantly reduced glucose, glycated haemoglobin (HbA1c), TC, LDL, TG, liver fat content, BMI, aspartate aminotransferase (AST), alanine aminotransferase (ALT) compared with the placebo [134]. All the changes seen may be due to the loss of weight seen in the curcumin group (over 2 kg difference over 8 weeks), which may be caused by nausea and anorexia, which caused 3 dropouts as well. Ultrasound is not a reliable method amount of liver fat. Large changes in HbA1c were seen in both groups. In a randomised double-blind, placebo-controlled trial of 100 overweight/obese subjects with T2DM (average age: 54.72 ± 8.34 years; BMI ≥ 24.0; curcuminoids (300 mg/day; *n* = 50) supplementation for 12 weeks significantly reduced fasting glucose, HbA1c and homeostasis model assessment insulin resistance (HOMA-IR) with decreased levels of serum free fatty acids (FFAs) and TG, and increased lipoprotein lipase (LPL) activity compared with a placebo. However, levels of TC, LDL- C, HDL- C, Apo A-I or Apo B did not differ. Anthropometric variables such as body weight and waist and hip circumferences were also not altered on curcuminoids supplementation compared with a placebo [135].

In a randomised, double-blind, placebo-controlled trial of prediabetic subjects (*n* = 237), curcumin treatment (a total of 6 capsules/day—250 mg curcuminoid/capsule) significantly decreased HbA1c, fasting glucose and OGTT at 3, 6, and 9 months compared with a placebo (*p* < 0.01) and reduced the diagnosis of T2DM from 16.9% to 0%. At 3 months, curcumin treatment significantly increased HOMA-β (*p* < 0.01) compared with a placebo. At 9 months, C-peptide and insulin were significantly attenuated with curcumin treatment compared with a placebo (*p* < 0.05). Moreover, curcumin treatment significantly reduced HOMA-IR at 6 and 9 months and elevated adiponectin at 9 months compared with a placebo [136]. Curcumin treatment significantly decreased body weight by 6.2 kg at 9 months compared with a placebo which could account for all of the observations on glucose and HbA1c. AST, ALT, creatinine, bone mineral density and waist circumference were not altered with curcumin treatment compared with a placebo. This clinical study supports the preventive effect of curcumin on the development of T2DM in individuals with prediabetes [136].

Adiponectin acts as an insulin sensitiser to suppress hepatic gluconeogenesis via AMP-activated protein kinase (AMPK)-dependent and -independent pathways. It stimulates fatty acid oxidation predominantly in skeletal muscle to activate glucose uptake [137]. Increased adiponectin levels were observed with curcumin supplementation (1 g/day) for 6 weeks in subjects with metabolic syndrome compared with a curcumin-phospholipid complex group or a placebo group, whereas no differences in BMI, body weight, waist circumference, fasting blood glucose, fat (%) were observed compared with a curcumin-phospholipid complex group or a placebo group [138].

In a randomised double-blind, placebo-controlled design, subjects with T2DM (mean age 59 ± 10.6 years; *n* = 107) were treated with 250 mg of curcumin (3 times/day) for 6 months. Curcumin treatment increased adiponectin and decreased leptin levels compared with the placebo. Pulse wave velocity (PWV), HOMA-IR, TG, uric acid, abdominal obesity (visceral fat and total body fat) were significantly reduced with curcumin treatment compare with the placebo [139].

In a randomised crossover study, 11 healthy subjects supplemented with turmeric (2.8 g/day) for 4 weeks showed no changes in fasting plasma glucose, TC and TG compared with the control (water only) [140]. In a crossover design, 14 healthy subjects who consumed 6 g of encapsulated turmeric with a standard 75 g oral glucose tolerance test (OGTT) showed increased insulin responses with no difference in postprandial glucose responses compared with the reference OGTT with placebo capsules [141].

The effect of curcumin on glucose control is inconclusive, as five RCTs [57,134,135,136,139] showed positive effects and ten RCTs [80,81,108,138,140,142,143,144,145,146] showed no effect.

#### Potential Mechanisms of Curcumin

On the other hand, in vitro and high-dose animal studies supports the glucose lowering effects of curcumin. A high-fat diet (HFD) was given to male Wistar rats (7 weeks of age) for 6 weeks, and then diabetes was induced by streptozotocin injection (30 mg/kg body weight). Three different concentrations of curcumin (50, 150, or 250 mg/kg body weight) were used for 7 weeks. Curcumin (especially, 150 mg/kg body weight) significantly improved glucose and insulin tolerance compared with normal control rats [147]. In addition, in the in vitro study, L6 myotube cells were treated with different concentrations of curcumin (5, 10, 20, or 40 μm/L) in the presence of palmitate (0.25 mM/L). The 2-deoxy-[3H] D-glucose uptake was enhanced by curcumin in a dose dependent manner and glucose transporter 4 (GLUT4) protein expression increased on the cell membrane of L6 myotubes [147].

C57BL/KsJ-db/db mice and their age-matched lean non-diabetic db/+ mice were fed a diet with curcumin or without curcumin (0.02%, wt/wt) for 6 weeks. In db/db mice, curcumin decreased glucose and HbA1c, improved HOMA-IR and glucose tolerance as assessed by intraperitoneal glucose tolerance test (IPGTT) and increased insulin levels. Curcumin did not alter glucose tolerance and insulin levels in db/+ mice. Curcumin increased hepatic glucokinase (GK) activity and suppressed the elevation of hepatic gluconeogenic enzyme activities, glucose-6-phosphatase (G6Pase) and phosphoenolpyruvate carboxykinase (PEPCK) in db/db mice. Curcumin increased glycogen storage in the liver in the db/db mice, while curcumin did not alter hepatic GK, P6Pase, PEPCK and glycogen content in non-diabetic db/+mice. These findings indicate that curcumin can lower glucose levels in db/db mice [93].

AMPK plays a key role in glucose and fatty acid metabolism [148]. Its roles in activation of catabolic processes aimed at ATP production (e.g., glucose uptake, glycolysis, fatty acid uptake, and β-oxidation) and inhibition of anabolic pathways (e.g., synthesis of glycogen, proteins, fatty acids, and cholesterol) have been well documented [149].

Curcumin treatment (10–20 µm/L, 10 µm/L, or 15, 30 and 60 mg/kg body weight) elevated insulin sensitivity by increasing insulin receptor substrate-1 (IRS1) protein, Akt, extracellular signal-regulated protein kinases 1 and 2 (ERK1/2), p38 MAPK, and AMPK in C2C12 skeletal muscle cells [150], L6 myotube cells [151] and in the liver of fructose-fed rats [152]. Curcumin (10 μm/L) also increased insulin sensitivity in skeletal muscle by promoting oxidation of glucose and fatty acid mediated in part through serine–threonine liver kinase B1 (LKB1)-AMPK pathway [147].

Curcumin (10 μm/L) improved insulin secretion by activating phosphoinositide 3–kinase (PI3K) or Akt, inhibiting forkhead box protein O1 (FoxO1), alleviating endoplasmic reticulum (ER) stress and enhancing mitochondrial survival from lipotoxicity in palmitate-treated MIN 6 pancreatic β cells [153].

Curcumin (2–10 μm/L) lowered glucose by stimulating β-cell function by activating the volume-regulated anion channel enhancing insulin secretion in rat pancreatic β-cells [154]. Curcumin (1–100 pmol/L) also enhanced pancreatic β-cell function acting as a cyclic nucleotide phosphodiesterase (PDE) inhibitor. Curcumin treatment downregulated expression of PDEs (enzymes which convert cyclic AMP and cyclic GMP into 5′ AMP and 5′-GMP), leading to elevated intracellular cAMP levels with improved insulin release from pancreatic β-cells [155].

In high-fat diet-induced obese and leptin-deficient ob/ob male C57BL/6J mice, the improved glucose control following curcumin treatment (3% dietary curcumin) was associated with anti-inflammatory effects of curcumin by decreasing macrophage infiltration into adipocytes, and by increasing adiponectin production in white adipocytes, by decreasing ER stress (elevated ER stress in adipocytes and hepatocytes is related to obesity) and perigonadal fat expression of Sirtuin 1 (SIRT1), heat shock proteins (HSP70), HSP90 and Foxo1 and decreasing NF-κB activity in liver [156]. SIRT1 is a regulator of adiponectin transcription through the activation of Foxo1 and Foxo1 and C/EBPalpha interaction in adipose tissue [157]. SIRT1 plays a role in glucose homeostasis, inducing hepatic glucose production through the deacetylation of PGC-1α and by repressing peroxisome proliferator-activated receptor gamma (PPAR γ) [158,159].

Curcumin supplementation (15, 30 and 60 mg/kg) increased IRS1 resulting in improved insulin sensitivity via increased expression of PPAR γ and Akt and ERK1/2 in liver of fructose-fed rats [152].

### 3.4. Lipid Homeostasis

Seven RCTs [45,68,100,134,135,139,143] showed positive lipid profile changes, while ten RCTs [57,79,80,81,108,140,142,145,160,161] showed no effect. Therefore, evidence that curcumin is beneficial is still lacking.

A further meta-analysis of RCTs also showed no effect of curcumin on lipid profiles [162]. According to a 2017 position paper from an International Lipid Expert Panel, the lipid lowering effect of curcumin in human intervention studies is inconsistent, but several recent interventions report favourable effects on lipid profiles [134,143,146,163,164].

In a randomised double-blind, placebo-controlled parallel study of 117 subjects with metabolic syndrome (aged 25–75 years), supplementation of 1000 mg/day of curcuminoids and piperine (bioperine^®^ Sami Labs LTD, Bangalore, Karnataka, India) (100:1 ratio combination) for 8 weeks significantly lowered LDL-C, non-HDL-C, TC, TG and lipoprotein(a) [Lp(a)] and increased HDL-C, compared with the placebo. The lipid changes were significant after adjustment for baseline of BMI and lipids. No change in small dense LDL (sdLDL) was observed [163].

In a randomised placebo-controlled study of 87 subjects with non-alcoholic fatty liver disease, 1000 mg/day of curcumin plus dietary and lifestyle intervention for 8 weeks significantly decreased TC, non-HDL-C, LDL-C, TG and uric acid compared with placebo. No differences in HbA1c, fasting glucose, insulin, HOMA-IR, HOMA-β, quantitative insulin sensitivity check index (QUICKI) were seen compared with placebo [146].

In a randomised, double-blind, placebo-controlled trial in 70 subjects with T2DM, nano-curcumin (80 mg/day) for 3 months significantly reduced HbA1C, fasting blood glucose, TG, and BMI before and after the treatment [57].

In a randomised, double-blind, placebo-controlled trial, 33 subjects aged 40–60 years with metabolic syndrome (mean BMI: 30.06 ± 4.12 kg/m^2^) were supplemented with 630 mg of curcumin extract (95% curcuminoids, including curcumin, demethoxycurcumin, and bisdemethoxycurcumin) three times per day for 12 weeks. In comparison with the placebo, LDL-C significantly decreased with curcumin supplementation with no changes in weight, BMI, fasting glucose, HbA1C, TG, TC, very low-density lipoprotein (VLDL), HDL-C, Non-HDL-C and T-Chol/HDL-C ratio [143].

In a randomised, double-blind, placebo-controlled, parallel study investigating the effect of curcumin (400 mg/day as Longvida^®^) on cognition, mood and biomarkers in 60 elderly subjects (mean age: 68.5 years) for 4 weeks significantly lowered TC and LDL-C with the significant improvement memory and mood compared with the placebo [68].

In a randomised double-blind, placebo-controlled crossover trial, 30 subjects aged 18–65 years who were not taking lipid-lowering agent, as well as who had any conditions including BMI ≥ 30 kg/m^2^ or 2 risk factors (except for T2DM) for coronary heart disease (CHD) or ≥ 2 risk factors (except for T2DM) for CHD and 130 mg/dL < LDL-C < 160 mg/dL, were supplemented with curcuminoids (1 g/day) for 30 days. Curcuminoids supplementation decreased TG levels with no differences, LDL-C, HDL-C and CRP. Anthropometric variables such as body weight, BMI, waist circumference, arm circumference, and fat percentage also were not altered with curcuminoids supplementation compared with the placebo [100].

In a randomised double-blind, controlled trial, subjects with acute coronary syndrome, curcumin ingestion (45–180 mg/day) for 1 year showed no changes in lipids [145]. In a randomised, double-blind study of 36 elderly subjects (mean age: 73.4 ± 8.8 years), either 4 g/day or 1 g/day of curcumin supplementation did not significantly alter TG, LDL-C and HDL-C over 1 month or 6 months compared with a control [160]. In a double-blind, randomised, placebo-controlled, 2 × 2 factorial trial, 70 hypercholesterolemia subjects (mean fasting TC: 6.57 ± 0.13 mM/L) were randomised to either curcumin (200 mg/day: Meriva ^®^ Indena) plus phytosterols (2 g/day; *n* = 17) or curcumin (200 mg/day; *n* = 18) or phytosterols (2 g/day; *n* = 17) or placebo (*n* = 18) for 4 weeks. Phytosterol group and curcumin plus phytosterol group showed significant reductions in TC, LDL-C and TC: HDL-C compared with the baseline, whereas the curcumin group did not significantly alter TC and LDL-C. HDL-C and TG were not altered in any group [161].

#### Potential Mechanisms of Actions of Curcumin on Lipids

In vitro [165,166] and animal studies [93,152,167,168,169,170,171,172,173,174] showed improved lipid profiles following curcumin treatment.

In the human hepatoma cell line (HepG2), a potential hypocholesterolemic effect of curcumin (0.02% wt/wt) was observed with elevated gene expressions of the LDL-receptor. Curcumin (80 mg/kg) has been shown to downregulate fatty acid synthase (FAS—related to increased plasma lipid levels) and increase skeletal muscle LPL [93,167].

Statins are lipid-lowering medications known as HMG-CoA reductase inhibitors. Curcumin (0.02% wt/wt) has been shown to decrease HMG-CoA reductase activity leading to reduced plasma and hepatic cholesterol levels [93,175].

In animals fed a high-fat diet, curcumin treatment (0.05 g/100 g diet) has been shown to decrease hepatic acyl-CoA: cholesterol acyltransferase (ACAT) which may decrease intestinal cholesterol uptake and transport in the intestine [93,172].

Curcumin treatment (0.5% dietary curcumin or 0.1% wt/wt) also has been shown to increase hepatic cholesterol 7 α-hydroxylase (CYP7A) which is a rate-limiting enzyme responsible for the biosynthesis of bile acid from cholesterol [171,176,177].

Activated AMPK suppresses fatty acid synthesis in the liver by suppressing liver X receptor α (LXR α) -dependent sterol regulatory element-binding protein [(SREBP)-1c—a major gene transcription factor for hepatic lipogenesis] [178,179]. Elevated hepatic lipogenesis is associated with insulin resistance [178,179]. In the liver, curcumin (4 g/kg diet or 0.02% wt/wt) suppressed SREBP1c, and the carbohydrate response element-binding protein (ChREBP) [98] and upregulated LXR α to decrease TG levels [175].

A cholesterol transporter, ATP-binding cassette A1 (ABCA1) transporter acts as cholesterol efflux regulatory protein. Curcumin (5, 10 and 20 μg/mL) has been shown to promote cholesterol efflux from adipocytes via PPAR γ-LXR α-ABCA1 pathway (activation of PPAR γ, LXR α and ABCA1) [180].

Ezetimibe, known as a Niemann-Pick C1 Like 1 (NPC1L1) inhibitor, is a cholesterol-lowering medication which reduces cholesterol absorption in the small intestine. Curcumin treatment (50 μm/L or 25–100 μm/L) in Caco-2 cells inhibited NPC1L1 through the inhibition of SREBP-2 [181,182].

Curcumin treatment (100 or 400 mg/kg body weight) has been shown to decrease serum TG and fetuin-A (α2-Heremans-Schmid glycoprotein) levels in liver of rats fed a high-fat diet [174]. Fetuin-A produced in the liver is associated with insulin resistance and fatty liver [183,184].

### 3.5. Weight Control

In a systematic review [185] of 8 RCTs [46,81,100,134,135,139,143,186], 3 studies [46,134,139] showed a favourable effect on weight control while 5 studies [81,100,135,143,186] showed no effects [185]. Moreover, RCTs [136,138,142] included in this review showed no effect, and RCTs [45,57] showed a positive effect on weight control. Given the findings from limited numbers of RCTs [45,46,57,81,100,134,135,136,138,139,142,143,186], curcumin at usual doses is unlikely to contribute to weight control.

In a randomised double-blind, placebo-controlled trial of subjects with T2DM, curcuminoids (1000 mg/day combined with piperine 10 mg/day) for 12 weeks significantly decreased body weight, BMI, TC and Lp(a) and increased HDL-C compared with a placebo. However, curcumin did not alter TG and LDL-C compared with the placebo [45].

In a randomised parallel trial, overweight subjects with metabolic syndrome (mean BMI 25–29.9 kg/m2; mean age 39.1 ± 16.8 years; *n* = 44) who adhered to a diet plus lifestyle intervention for 30 days (adherence rate > 80% and a weight loss < 2%) underwent either curcumin treatment (800 mg twice per day; *n* = 22) plus lifestyle intervention or phosphatidylserine plus lifestyle intervention for 4 weeks. Significant reductions in body weight, body fat, waistline and BMI were observed with curcumin treatment compared with the phosphatidylserine group [46].

On the other hand, in a randomised, double-blind, placebo-controlled, crossover study, obese subjects (mean BMI: 33.95 ± 3.81) who were treated with curcuminoids (1 g/day) for 30 days showed no differences in BMI and weight compared with the placebo [186].

#### Potential Mechanisms

Curcumin (80 mg/kg diet or 500 mg/kg diet or 1.5 g/kg diet or 4 g/kg diet or HFD containing 0.15% curcumin) led to reduced body weight and/or body fat in HFD-fed mice [98,156,167,168,169]. Curcumin (5, 10, 20 μm/L) has been documented to decrease palmitate-induced insulin resistance in 3T3-L1 adipocytes [126]. Curcumin (0-100 μm/L) has also been shown to inhibit glucose transport in 3T3-L1 adipocytes [187,188,189]. Curcumin (10, 25, 50, and 75 µm/L) induced insulin resistance by suppressing insulin-stimulated protein kinase B (Akt)/GLUT4 signalling with activation of autophagy in 3T3-L1 adipocytes [189].

Stearoyl-coenzyme A desaturase 1 (SCD-1) is a rate enzyme in the synthesis of unsaturated fatty acids [190]. Upregulation of SCD-1 may be associated with obesity, insulin resistance and atherosclerosis [191]. Curcumin (80 mg/kg) treatment for 12 weeks in HFD-induced obese mice has been shown to downregulate the expression of SCD-1 in brown adipose tissue and white adipose tissue [167].

The browning effect of white fat by curcumin has been reported in several studies [192,193,194]. Brown adipose tissues generate heat from fat through uncoupling proteins (UCP1) present in the mitochondrial inner membrane. Brown adipose tissues are known to play a critical role in non-shivering thermogenesis against cold [195]. Brown adipose tissues can be derived from white adipose tissues with stimuli including cold, and adrenergic compounds [196,197,198,199,200,201]. Thermogenic gene expression (e.g., UCP1, PGC1 α, Prdm16, Dio2, PPAR γ, Cidea, Elovl3, Nrf1, mtTfa, and ATPsyn) and mitochondrial biogenesis are increased with the browning of white adipose tissue. Curcumin (80 mg/kg) treatment for 12 weeks in HFD-induced obese mice has been shown to upregulate mRNA expressions of UCP1 in brown adipose tissue and white adipose tissue [167].

Norepinephrine binding to beta 3 adrenoreceptors (β3AR) present in white adipose tissues are known to exert a key role in the browning of white adipose tissues [202]. Curcumin treatment (50 or 100 mg/kg) in C57BL/6J mice elevated plasma norepinephrine levels and upregulated β3AR gene expression in inguinal white adipose tissues and induced browning with the decreased body weight and fat accumulation compared with control mice [192].

Curcumin (1–20 μm/L) induced fat browning through AMPK-mediated pathway in 3T3-L1 and primary white adipocytes [193]. Curcumin (40 and 80 mg/kg) decreased white and brown adipocyte diameters and insulin resistance caused by a high fat diet via inhibition of SREBPs [167].

Curcumin (25 µm/L) also exerts an anti-adipogenic effect via stimulating the Wnt/β-catenin signalling pathway with stimulation of downstream targets such as c-Myc and cyclin D1, and by inhibiting glycogen synthase kinase-3 beta (GSK-3β) and Axin, consequently resulting in downregulation of JNK phosphorylation. Curcumin treatment in 3T3-L1 cells suppressed adipocyte differentiation by suppressing adipogenic transcription factors (C/EBPα, PPAR γ and C/EBPα) and their downstream factors (SREBP-1 and FASN) [203].

### 3.6. Gut Microbiome Changes

Lifestyle including dietary pattern, exercise, sleep and environmental factors can affect the gut microbiota [204,205,206,207]. Gut microbial dysbiosis is associated with obesity, metabolic syndrome, diabetes, CVD and neurodegenerative diseases [208,209,210,211,212].

Very limited studies showed that curcumin favourably modified the gut microbiota composition [213,214,215,216,217]. HFD-fed rats administrated with curcumin showed enhanced diversity of bacterial strains. A HFD increased metabolic endotoxemia and gut inflammation, which decreased with curcumin administration (200 mg/kg) [217].

Gut microbial dysbiosis appears to increase gut permeability, leading to an increased inflammatory response [218]. A positive association between a high-fructose diet and/or a high-fat diet and increased gut permeability has been reported [217,218,219,220].

In vitro, LPS increases IL-1 β which can activate p38 MAP kinases and subsequently myosin light chain kinases (MLCK). The phosphorylation of myosin light chains can increase gut permeability. Curcumin treatment decreased LPS-induced release of IL-1 β from intestinal epithelial cells and intestinal macrophages. Curcumin (5 µm/L) also suppressed p38 MAPK activation by IL-1 β and myosin light chain kinase in intestinal epithelial cells [221].

Animal studies [222,223,224,225] also showed improvement of gut permeability with the curcumin treatment. A mouse model fed a Western diet for 16 weeks exhibited elevated intestinal permeability [224]. Curcumin (5 µm/L) decreased plasma LPS levels and increased intestinal alkaline phosphatase activity and tight junction protein expressions (ZO-1 and claudin-1) [224].

Mitogen-activated protein kinase phosphatase 1 (MKP-1) exerts an essential role in dephosphorylating MAPK and inactivating ERK, JNK and p38 in response to stress [226,227]. Curcumin (100 mg/kg) decreased the impairment of intestinal mucosa barrier by methotrexate in rats through the activation of MKP-1 and suppression of p38 and NF-kB [222]. Therefore, curcumin appears to reduce gut permeability induced by external dietary factors (e.g., a western diet) or exogenous injury by altering signal pathways, consequently leading to the prevention of a chronic inflammatory state [228].

### 3.7. Endothelial Function

Curcumin has a protective effect on endothelial dysfunction [108,229,230,231,232,233,234]. The improved endothelial function on curcumin treatment is attributable to several mechanisms including hypoglycaemic [229] and hypolipidemic effects [229], and anti-inflammatory [235,236], anti-oxidant activities [229,230,232,233].

In a randomised, controlled, double-blind parallel study of 59 healthy subjects, curcumin supplementation (200 mg/day) for 8 weeks improved endothelial function as assessed by flow-mediated dilation (FMD) compared with placebo [237]. Sixty-seven subjects with T2DM who ingested NCB-02 (300 mg/day of curcumin) for 8 weeks showed significantly enhanced endothelial function (measured using digital plethysmography) with lower levels of MDA, ET-1, IL-6 and TNF-α compared with the baseline, but statistical comparison was not made with placebo. These beneficial effects of curcumin (NCB-02) on endothelial function through anti-inflammatory and antioxidant actions are comparable to those of atorvastatin (10 mg/day). However, in comparison with the baseline, NCB-02 did not significantly alter fasting glucose, HbA1c, TC, LDL-C HDL-C and TG, while atorvastatin significantly lowered TC, LDL-C and TG [108].

In a double-blind, parallel, randomised study of healthy middle-aged and older adults (45–74 years) curcumin supplementation (2000 mg/day Longvida^®^) for 12 weeks improved resistance artery endothelial function with enhanced forearm blood flow to brachial artery infusion of acetylcholine (FBFACh) when infused with vitamin C following curcumin compared with baseline (but not placebo in any variable). Curcumin also increased brachial artery FMD. Curcumin reversed the decrease in FBFACh from the nitric oxide synthase inhibitor, NG monomethyl-L-arginine. Curcumin did not alter the levels of adiponectin, leptin, insulin, HOMA-IR, oxidised LDL-C, total antioxidant status, GPx, IL-6, TNF-α, cortisol, epinephrine, norepinephrine and endothelin-1 (ET-1) compared with baseline or placebo [144].

Curcumin treatment (30 and 300 mg/kg) in streptozotocin-induced diabetic rats reduced vascular superoxide anion (O2●−) production and inhibited vascular protein kinase C (PKC-bII) resulting in improved endothelial function [232]. Vascular endothelial cell damage resulting from oxidative stress can be protected by curcumin treatment via autophagy activation. In human umbilical vein endothelial cells (HUVECs) under the condition of oxidative stress induced by hydrogen peroxide H_2_O_2_, curcumin treatment (1, 5 and 10 μm/L) increased microtubule-associated protein 1 light chain 3-II (LC3-II—an autophagosomal marker) in a dose-dependent manner.

Curcumin (1, 5 and 10 μm/L) protects vascular endothelial cells through autophagic process by inhibiting the PtdIns3K-AKT-mTOR signalling pathway and FOXO1 (a mediator of autophagy) nuclear localisation and FOXO1-related proteins [230].

Curcumin (0.25–2.5 μm/L) also protects vascular endothelial cells by trapping methylglyoxal (MGO—a major reactive dicarbonyl compound) and reducing Nε-(carboxymethyl) lysine (CML) formation in HUVECs. This protective effect is responsible for directly trapping MGO by curcumin [231].

Curcumin treatment (2.5, 5, 10 and 20 µm/L) of HUVECs and lymphocytes (Jurkat cells) exposed to either high glucose or advanced glycation end products (AGEs) restored transmembrane potential. Curcumin treatment decreased membrane fluidity in AGE-exposed Jurkat cells or glucose-exposed Jurkat cells. In addition, curcumin treatment inhibited MCP-1 release from Jurkat cells and HUVECs exposed to AGEs or glucose showing an anti-inflammatory action [238].

Lectin-like oxidised LDL receptor-1 (LOX-1), a receptor expressed in vascular cells for oxidised LDL, contributes to the pathogenesis of diabetic atherosclerosis [239]. As the glucose concentrations (5.6 to 30 mM/L) increased, expressions of LOX-1 gene and protein increase in human monocyte-derived macrophage (MDMs) [236]. The high concentration of glucose led to increased formation of foam cells in macrophage [240]. In a vitro study curcumin (10 μm/L) via activated protein-1 (AP-1—a transcription factor [241]) inhibition appeared to suppress the formation of macrophage-derived foam cells stimulated with high glucose levels via the LOX-1–dependent pathway [236]. Curcumin (5 μm/L) can prevent macrophages via LXRα dependent AMPK-signalling pathways in the human THP-1 cell line from being transformed into foam cells leading to the prevention of atherosclerosis [165].

Early growth response-1 gene product (Egr-1) is a pathophysiological transcription factor which induces the activity of plasminogen activator inhibitor type-1 (PAI-1). Elevated PAI-1 is related to increased insulin resistance. Curcumin (0–40 µm/L) downregulated Egr-1 protein in HUVECs [235].

Reduced NO bioavailability (or reduced NO synthesis) is associated with endothelial dysfunction [242]. Studies [243,244,245] showed that curcumin upregulated eNOS to improve endothelial dysfunction in HUVECs when oxidative stress is induced by H2O2 [244] in 2K-1C hypertensive rats [243] and in A10 vascular smooth muscle cells [245].

Curcumin has shown cardioprotective effects [246,247]. Curcumin treatment (150 mg/kg or 100 µm/L) of diabetic rat hearts decreased oxidative DNA and protein damage by decreasing levels of endothelial nitric oxide synthase (eNOS) and inducible nitric oxide synthase (iNOS) via reduced NF-kB and AP-11 in both diabetic rat hearts and in microvascular endothelial cells induced by high glucose. [246]. A vasoconstrictor, ET-1 levels increased with curcumin treatment in diabetic rat hearts and microvascular endothelial cells but ET-1 levels in the kidneys and the retina decreased, indicating that curcumin acts differently on organs [246].

Curcumin treatment (5, 10 and 30 μm/L) inhibited inflammatory cytokines (MCP-1, TNF-α, toll-like receptors (TLRs) and iNOS) and NO generation in vascular smooth muscle cells of rats treated with LPS through the downregulation of TLR4-MAPK/NF-kB pathways related with NADPH-mediated intracellular ROS generation [248].

### 3.8. Neurodegenerative Diseases

Diabetes mellitus enhances the risk of dementia. The impairments in glucose metabolism, insulin signalling, insulin sensitivity and lipid metabolism, as well as increases in inflammation and oxidative stress in central nerve and peripheral system, contribute to the risk of Alzheimer’s disease (AD) [249,250,251,252].

The etiology of AD includes accumulation of fibrillar amyloid-β (Aβ) peptides (amyloid plaques), decreased Aβ degradation enzymes, Aβ oligomer-promoted synaptic dysfunction, neurotoxic mediators from glial cells, apoE4 (lipid transport protein)-enhanced Aβ deposits, impaired mitochondrial function, mis-localised tau protein from axons to neuronal soma and dendrites, increased neurofibrillary tangles (NFTs—comprised of hyperphosphorylated tau) formation, self-assembled α-synuclein, vascular abnormalities, impaired supply of nutrients, impaired metabolic by-product removals, as well as activated glial cells [252]. Loss of neurons in certain brain areas such as pyramidal cells in lamina II of the entorhinal cortex and in the CA1 region of the hippocampus are responsible for early AD pathology [253,254].

Moreover, the accumulation of advanced glycation end products (AGEs) and the receptor for advanced glycation end products (RAGE) are associated with the T2DM, CVD, degenerative disease and ageing [255,256,257,258]. The increased AGE binding to microglia, neurons and vascular endothelia cells stimulates inflammatory actions and Aβ influx, which in turn leads to neuronal disfunction, cognitive decline and brain damage [259,260].

Aβ oligomers in cultured hippocampal neurons lead to phosphorylation of tau and inhibition of IRS-1 (Ser616) through the activation of c-Jun N-terminal kinase signalling, while curcumin treatment of triple transgenic -AD mice on a high-fat diet showed reductions in phosphorylated JNK, IRS-1, and tau in their brain [261].

Islet amyloid polypeptide (IAPP) or amylin is a hormone consisting of 37 amino acid residues which is co-released with insulin from pancreatic β cells [262]. The accumulation of amyloid as a consequence of the IAPP misfolding is associated with T2DM, AD and Parkinson’s disease [263,264]. Curcumin inhibited the self-assembly of IAPP [265,266].

In the hippocampal CA1 area of the brain of AD mice (PS1dE9 double transgenic mice model), curcumin upregulated the expressions of GLUT1 and GLUT3 indicating an improvement in cerebral glucose uptake. Curcumin also stimulated insulin-like growth factor (IGF)-1R, IRS-2, phosphoinositide 3-kinase (PI3K), p-PI3K, Akt and p-Akt protein, and suppressed IR and IRS-1 which implicates the improvement in insulin signalling pathways [267]. In addition, curcumin enhanced spatial memory and learning as assessed by the water maze behaviour test [268]. Aβ-derived diffusible ligands (ADDLs) are known to dysfunction insulin signalling [269,270,271] and act as neurotoxins [272] in AD. Curcumin treatment showed decreases in Aβ40, Aβ42, ADDLs and γ-secretase [presenilin (PS2)] expression, as well as increases in Aβ degradation enzymes such as insulin-degrading enzymes and neprilysin in the hippocampus CA1 region. The changes in these molecules are related with improved behaviour functions [268].

A recent RCT indicated that curcumin could reduce amyloid and tau accumulation in a certain brain region leading to the improvement memory and attention. Non-demented adults (aged 51–84 years; *n* = 21) who ingested curcumin (Theracurmin^®^ containing 90 mg of curcumin twice daily) for 18 months showed the enhanced memory and attention compared with a placebo. Amyloid and tau accumulation on brain were assessed by 2-(1-[71] ethylidene)malononitrile positron emission tomography (FDDNP-PET). Curcumin significantly lowered FDDNP binding in the amygdala (ES  =  −0.41, *p*  =  0.04) compared with a placebo (ES  =  0.08, *p*  =  0.6; between-group: ES  =  0.48, *p*  =  0.07) [71].

An AD brain shows an overexpression of PI3K/Akt/mTOR signalling, which is associated with insulin resistance and the pathology of Aβ and tau [273,274]. Curcumin decreased cognitive impairment as assessed by Morris water maze test, inhibited Aβ generation, and suppressed PI3K, phosphorylated Akt and mammalian target of rapamycin (mTOR—Akt regulating cell growth, proliferation, survival, angiogenesis, as well as autophagy [275]) leading to stimulated autophagy, in APP/PS1 double transgenic mice (an AD mice model) [276].

The modification of synapse structure and function is associated with the development of AD. The decreases in synapse-related proteins, including PSD95 and Shank1, contribute to the pathology of AD. Curcumin treatment improved the quantity and structure of the synapse by facilitating the PSD95 and Shank1 in the hippocampal CA1 regions of the APPswe/PS1dE9 double transgenic mice [277]. Curcumin treatment improved synaptic plasticity and neurogenesis resulting in improved memory function [278,279,280].

Brain-derived neurotrophic factor (BDNF) is a protein which stimulates neurogenesis, synaptic plasticity and memory in hippocampus and frontal cortex (FC) [281]. Decreased levels of BDNF are reported in obese and diabetic conditions [282,283,284,285]. Franco-Robles et al. 2014 [142] conducted both animal and human studies to investigate effects of curcumin on BDNF levels in the hippocampus and FC of diabetic db/db mice and in sera of obese subjects. Compared to baseline, curcumin treatment (50 mg/kg daily) for 8 weeks normalised BDNF levels in the hippocampus and FC of diabetic db/db mice. However, nondiabetic obese human subjects who consumed curcumin (500 and 750 mg/day) for 12 weeks did not have altered BDNF levels. Curcumin (500 mg) significantly decreased LDL-C in weeks 2 and 12. Curcumin (500 mg) in weeks 6 and 12 significantly decreased TBARS and oxLDL. Curcumin (500 mg and 750 mg) significantly lowered protein carbonyls levels in week 2, 6 and 12. Curcumin (500 mg and 750 mg) did not alter body weight, BMI, fat, glucose, TC, TG, HDL-C, VLDL and uric acid during the study period [142].

Membrane integrity is important to mainting the normal function of mitochondria and synapse in brain [286,287]. Curcumin facilitated the DHA biosynthesis in liver and DHA accumulation in the brain indicating that curcumin (1–10, 20, or 40 µm/L) can improve cell membrane integrity in the brain, consequently leading to the improvement of neurodegenerative disease by enhancing mitochondria and synaptic function [288,289].

Inflammation is a key player in the development of neurodegenerative diseases [290]. Microglial cells are resident macrophages that play a crucial role in innate immune regulation and brain homeostasis [291,292,293]. COXs participate in PGs formation [294]. The expression of COXs occurs when microglial cells and astrocytes are stimulated by inflammation [295]. Curcumin treatment (4–20 µm/L) inhibited LPS-activated COX-2 gene expression and pro-inflammatory cytokines through the suppression of NF-kB and Activator Protein 1 (AP-1) in BV2 microglial cell [296,297]. Curcumin treatment (20 µm/L) suppressed migration of microglia in LPS-activated BV-2 cells with the inhibition of IL-4, PPAR-α, TLR 2, Prostaglandin-endoperoxide synthase 2 and NO synthase 2, indicating an anti-inflammatory and neuroprotective effects of curcumin [298].

## 4. Conclusions

**Figure 2:**
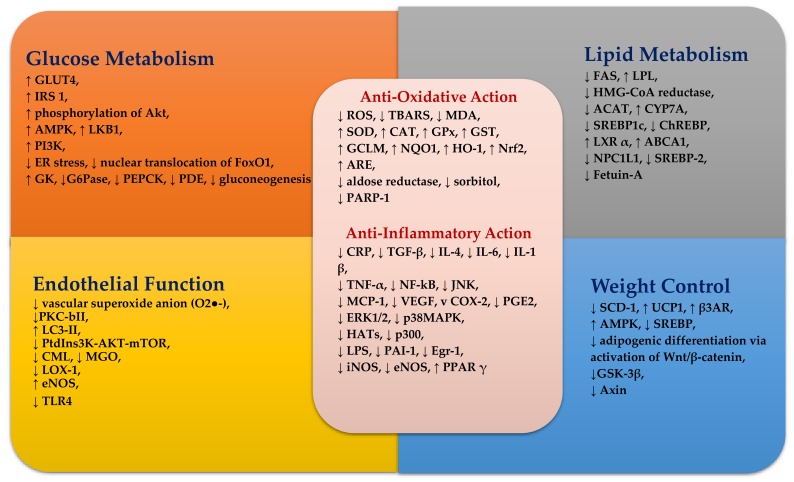
The summary of potential mechanisms linking curcumin metabolites to improved glucose, lipid metabolism, antioxidative action, anti-inflammatory action and endothelial function. Refer to the text for more details. ↑—increase; ↓—decrease; ABCA1—ATP-binding cassette A1; ACAT—acyl-CoA cholesterol acyltransferase; Akt—serine/threonine kinase; AMPK—5′ adenosine monophosphate–activated protein kinase; ARE—antioxidant-responsive element; β3AR—β3-adrenergic receptor; CAT—catalase; ChREBP—carbohydrate response element-binding protein; CML—Nε-(carboxymethyl) lysine; COX-2—cyclo-oxygenase 2; CRP—C–reactive protein; CYP7A—cholesterol 7 α-hydroxylase; Egr-1—early growth response-1 gene product; eNOS—endothelial nitric oxide synthase; ER—endoplasmic reticulum; ERK1/2—extracellular signal-regulated protein kinases 1 and 2; FAS—fatty acid synthase; FOXO1—forkhead box protein O1; GCLM—γ-glutamyl-cysteine ligase; G6Pase—glucose–6–phosphatase; GK—glucokinase; GLUT4—glucose transporter 4; GPx—glutathione peroxidase; GSK-3β—glycogen synthase kinase-3 beta; GST—glutathione-S-transferase; HATs—histone acetylases; HMG-CoA reductase—3-Hydroxy-3-methylglutaryl-coenzyme A reductase; HO-1—heme oxygenase1; IL—interleukin; iNOS—inducible nitric oxide synthase; IRS1—insulin receptor substrate-1; JNK—Jun NH2-terminal kinase; LKB1—serine–threonine liver kinase B1; LOX-1—lectin-like oxidised LDL receptor; LPL—lipoprotein lipase; LPS—lipopolysaccharides; LXRα—liver X receptor alpha; MAPK—mitogen-activated protein kinase; MCP-1—monocyte chemoattractant protein 1; MDA—malondialdehyde; MGO—methylglyoxal; NF-kB—nuclear factor kappa B; mTOR—mammalian target of rapamycin; Nrf2—nuclear factor erythroid 2–related factor 2; NQO1—NAD(P)H dehydrogenase [quinone] 1; NPC1L1—Niemann-Pick C1 Like 1; PAI-1—plasminogen activator inhibitor type -1; PARP-1—poly ADP-ribose polymerase-1; PDE—phosphodiesterase; PEPCK—phosphoenolpyruvate carboxykinase; PGE2—prostaglandin E2; PI3K—phosphoinositide 3–kinase; PKC-Bii—protein kinase C; PPAR—peroxisome proliferator-activated receptor; ROS—reactive oxygen species; SCD-1—stearoyl-coenzyme A desaturase 1; SOD—superoxide dismutase; SREBP1c—sterol regulatory element-binding protein 1c; TBARS—thiobarbituric acid reactive substances; TGF-β—transforming growth factor beta; TLR4—toll-like receptors 4; TNFα—tumor necrosis factor α; UCP1—uncoupling protein 1; VEGF—vascular endothelial growth factor.

A summary of possible mechanisms of curcumin on glucose homeostasis, lipid metabolism, oxidative stress, inflammation and endothelial function is shown in Figure 2.

In conclusion, based on high-dose animal and in vitro studies, curcumin appears to be a promising therapeutic agent to decrease the risk of T2DM, CVD and neurodegenerative disease by improving glucose homeostasis, lipid metabolism, endothelial function and insulin signalling, and by inhibiting Aβ aggregation. These favourable effects of curcumin could be attributable to potent anti-oxidant and anti-inflammatory actions.

To avoid misinterpretation of results from in vitro studies, it should be noted that cells used in most of the in vitro studies were exposed to very high level of curcumin, i.e., 10 to 100 times greater than the circulating dose measured in plasma after consumption of curcumin supplements or curcumin-rich meal.

There is variability in curcumin metabolism between humans and animals. Thus, the results of in vitro and animal studies cannot be directly related to human physiology. The formula to convert animal doses into human doses for the determination of the equivalents of animal doses in human doses is as follows: human effective dose (mg/kg body weight) = animal dose (mg/kg) X animal km/human km. The correction factor (km) is calculated by dividing the average body weight (kg) of species to its body surface area (m^2^) [299]. Frequently, animal doses are well beyond normal human doses.

Curcumin is metabolised to curcumin glucuronide or curcumin sulphate by glucuronidase and sulfatase, respectively. The predominant curcumin glucuronides metabolites are terahydrocurcumin (THC) and hexahydrocurcumin (HHC). The minor curcumin metabolites are dihydroferulic acid and ferulic acid. These water-soluble metabolites are excreted through the urine [30]. Ninety-nine percent of plasma curcumin is glucuronide-conjugates. The maximum plasma concentrations of curcuminoid conjugates were observed within 1 h of oral administration in humans [25]. It is unclear whether curcumin metabolites are more or less bioactive than native curcumin [30,300,301,302].

Human studies at present are relatively unconvincing. So far, new curcumin formulations have been tested to assess an increase in bioavailability and efficacy in animal studies. A few clinical interventions have been conducted to see if the increased bioavailability with the new curcumin formulations is associated with cardiometabolic benefit, but no direct comparisons have been made with standard formulations.

## Figures and Tables

**Table 1:** Summary of curcumin human intervention studies.

Dose	Design	Subjects	Period	Effects	No Effects	Ref
1 g/day of curcuminoids (curcumin C3 Complex® Sami Labs LTD, Bangalore, Karnataka, India) combined with 10 mg/day of piperine	randomised double-blind, placebo-controlled	100 subjects with T2DM aged 18–65 years	12 weeks	↓ body weight, ↓ BMI, ↓ TC, ↓ Lp(a), ↑ HDL-C compared with the placebo	TG, LDL-C compared with the placebo	Panahi et al. 2017 [45]
800 mg × 2/day of curcumin-based product (enteric-coated and containing 800 mg/dose/die of 95% curcumin complexed with 20% phosphatidylserine and blended with 8 mg/dose/die of piperine) + lifestyle intervention, or 400 mg × 2 day of phosphatidylserine + lifestyle intervention	randomised parallel	44 overweight subjects with metabolic syndrome (mean BMI 25–29.9 kg/m^2^; mean age 39.1 ± 16.8 years)	4 weeks	↓ body weight, ↓ body fat, ↓ waistline, ↓ BMI compared with the phosphatidylserine group		Di Pierro et al. 2015 [46]
80 mg/day of nano- micelle curcumin (SinaCurcumin® Exir Nano Sina Co., Tehran, Tehran province, Iran)	randomised, double-blind, placebo-controlled	70 subjects with T2DM aged over 18 years	3 months	↓ HbA1_C_, ↓ fasting blood glucose, ↓ BMI compared with the placebo	TC, LDL-C, HDL-C compared with the placebo	Rahimi et al. 2016 [57]
400 mg /day of Longvida® (containing ~ 80 mg curcumin in a solid lipid formulation)	randomised, double-blind, placebo-controlled, parallel	60 elderly subjects (mean age: 68.5 years)	4 weeks	↓ TC, ↓ LDL-C, ↑ memory, ↑ mood compared with the placebo		Cox et al. 2015 [68]
80 mg/day of lipidated curcumin (Longvida®)		38 healthy middle-aged subjects (40–60 years old)	4 weeks		TC, LDL, HDL-C, CRP, TG, salivary amylase, salivary radical scavenging capacities, catalase, beta amyloid protein, sICAM, myeloperoxidase, nitric oxide, ALT	Disilvestro et al. 2012 [79]
500 mg/day of curcumin	open uncontrolled	14 subjects with T2DM	15 days		fasting glucose, insulin, C-peptide, TG, TC, HDL-C, LDL-C, ALT, AST, BUN	Yang et al. 2015 [80]
2.8 g/day of turmeric (~112 mg/day of curcumin)	randomised, double-blind, placebo-controlled, crossover	62 overweight/obese women aged over 40–75 years [mean (BMI) ≥ 34.5 ± 0.8 kg/m^2^] with CRP = 8.05 ± 1.33 mg/L	4 weeks		F2-iso-prostanes, oxidised LDL-C, CRP, IL-6, IL-8, IL-10, TNFα, IFNγ, IL-1β, IL-12p70, glucose, body weight, percent body fat, SBP, augmentation index	Nieman et al. 2012 [81]
1 g/day of curcuminoids (500 mg C3Complex® + 5 mg bioperine®)	randomised double-blind placebo-controlled crossover	30 subjects aged 18–65 years who were not taking lipid-lowering agent, as well as who had any conditions including BMI ≥ 30 kg/m^2^ or 2 risk factors (except for T2DM) for CHD or ≥ 2 risk factors (except for T2DM) for CHD and 130 mg/dL < LDL-C <160 mg/dL	30 days	↓ TG compared with the placebo	LDL-C, HDL-C, CRP. body weight, BMI, waist circumference, arm circumference, fat percentage	Mohammadi et al. 2013 [100]
300 mg/day of curcumin (NCB-02)	randomised, placebo-controlled, parallel	67 subjects with T2DM aged 21–80 years	8 weeks		fasting glucose, HbA1c, TC, LDL-C, HDL-C and TG endothelial function, MDA, ET-1, IL-6, TNF-α	Usharani et al. 2008 [108]
1 g/day of curcuminoids (500 mg C3Complex® + 5 mg bioperine®)	randomised, double-blind, crossover	30 obese subjects with BMI ≥ 30	4 weeks	↓ IL-4, ↓ IL-1 β, ↓ VEGF compared with the placebo	IL-2, IL-6, IL-8, IL-10, IFN γ, EGF, MCP-1	Ganjali et al. 2014 [111]
1 g/day of curcumin		117 subjects with metabolic syndrome	8 weeks	↓ TNF-α, ↓ IL-6, ↓ TGF-β, ↓MCP-1 compared with placebo		Panahi et al. 2016 [112]
500 mg/day of an amorphous dispersion curcumin formulation (comprising 70-mg curcuminoids)	randomised double-blind, placebo-controlled	77 subjects with NAFLD (mean age 46.37 ± 11.57 years; mean BMI 31.35 ± 5.67 kg/m^2^)	8 weeks	↓ glucose, ↓ HbA1c, ↓ TC, ↓ LDL, ↓ TG, ↓ liver fat, ↓ BMI, ↓ AST, ↓ ALT compared with the placebo		Rahmani et al. 2016 [134]
300 mg/day of curcuminoids	randomised double-blind, placebo-controlled	100 overweight/obese subjects with T2DM (average age: 54.72 ± 8.34 years; BMI ≥ 24.0	12 weeks	↓ fasting glucose, ↓ HbA1c, ↓ HOMA-IR, ↓ FFAs, ↓ TG, ↑ LPL compared with a placebo	TC, LDL- C, HDL- C, Apo A-I or Apo B, body weight, waist and hip circumferences	Na et al. 2013 [135]
1.5 g/day of curcuminoid	randomised, double-blind, placebo-controlled	237 prediabetic subjects	3, 6, 9 months	↓ HbA1c, ↓ fasting glucose, ↓ OGTT at 3, 6, and 9 months, ↓ diagnosis of T2DM, ↑ HOMA-β at 6 & 9 months, ↑ adiponectin at 9 months, ↓ C-peptide at 9 months, ↓ insulin at 9 months, ↓ body weight at 9 months compared with the placebo	AST, ALT, creatinine, bone mineral density, waist circumference	Chuengsamarn et al. 2012 [136]
1 g/day of phospholipidated curcumin (*n* = 40) equivalent to 200 mg/day of pure curcumin, or 1 g/day of unformulated curcumin (*n* = 40), or placebo (*n* = 40)	Randomised, double-blind, placebo-controlled	120 subjects with metabolic syndrome aged 18–65 years	6 weeks	↑ adiponectin in the unformulated curcumin group compared with the curcumin-phospholipid complex group or the placebo group	BMI, body weight, waist circumference, fasting blood glucose, fat (%) compared with a curcumin-phospholipid complex group or a placebo group	Salahshooh et al. 2017 [138]
750 mg/day of curcuminoid	randomised double-blind, placebo-controlled	subjects with T2DM (mean age 59 ± 10.6 years; *n* = 107	6 months	↑ adiponectin, ↓ leptin, ↓ PWV, ↓ HOMA-IR, ↓ TG, ↓ uric acid, ↓ visceral fat, ↓ total body fat compared with the placebo		Chuengsamarn et al. 2014 [139]
2.8 g/day of turmeric	randomised crossover	11 healthy subjects aged 21–38 years	4 weeks		fasting glucose, TC, TG	Tang et al. 2008 [140]
6 g of curcuma *C. longa* (turmeric)	crossover, acute	14 healthy subjects (mean age: 29 ± 1 years, BMI: 23.9 ± 2.7 kg/m^2^)		↑ insulin responses compared with the placebo	postprandial glucose responses	Wickenberg et al. 2010 [141]
500 and 750 mg/day of curcumin	randomised, single-blind, placebo-controlled	nondiabetic obese subjects	12 weeks		BDNF, body weight, BMI, fat, glucose, TC, LDL, TG, HDL-C, VLDL, uric acid, oxLDL, protein carbonyls	Franco-Robles et al. 2014 [142]
1890 mg/day of curcumin extract	randomised, double-blind, placebo-controlled	33 subjects aged over 40–60 years with metabolic syndrome (mean BMI: 30.06 ± 4.12 kg/m^2^)	12 weeks	↓ LDL-C compared with the placebo	weight, BMI, fasting glucose, HbA1C, TG, TC, VLDL, HDL-C, Non-HDL-C and T-Chol/HDL-C ratio	Yang et al. 2014 [143]
2 g/day of curcumin (Longvida®)	double-blind, parallel, randomised	39 healthy middle-aged and older adults (45–74 years)	12 weeks	Nil compared with placebo	adiponectin, leptin, insulin, HOMA-IR, oxidised LDL-C, total antioxidant status, GPx, IL-6, TNF-α, cortisol, ET-1 FMD resistance artery endothelial function (FBF_ACh_)	Santos-Parker et al. 2017 [144]
45–180 mg/day of curcumin	randomised double-blind controlled	75 subjects with acute coronary syndrome	1 year		TC, LDL-C, HDL-C, TG, fasting glucose, 2-hour glucose	Alwi et al. 2008 [145]
1000 mg/day of curcumin + dietary and lifestyle intervention	Randomised, placebo-controlled	87 subjects with NAFLD	8 weeks	↓ TC, ↓ non–HDL-C, ↓ LDL-C, ↓ TG ↓ uric acid compared with the placebo.	HbA1c, fasting glucose, insulin, HOMA-IR, HOMA-β, quantitative insulin sensitivity check index (QUICKI) compared with the placebo. insulin, HOMA-IR, HOMA-β. QUICKI within the group.	Panahi et al. 2016 [146]
4 g/day or 1 g/day of curcumin	randomised, double-blind, placebo-controlled	36 elderly subjects (mean age: 73.4 ± 8.8 years)	over 1 month or 6 months		TG, LDL-C, HDL-C	Baum et al. 2007 [160]
200 mg/day of curcumin (Meriva ®, Indena SpA, Viale Ortles, Milan, Italy) + 2 g/day of phytosterols (*n* = 17), or 200 mg/day of curcumin (*n* = 18), or 2 g/day of phytosterols (*n* = 17), or placebo (*n* = 18)	double-blind, randomised, placebo-controlled, 2 × 2 factorial trial	70 hypercholesterolemia subjects (mean fasting TC: 6.57 ± 0.13 mM/L) aged 18–70 years	4 weeks	↓ TC, ↓LDL-C and↓ TC: HDL-C within phytosterol group and within curcumin plus phytosterol group. No change with curcumin alone		Ferguson et al. 2018 [161]
1000 mg/day of curcuminoids and piperine (bioperine®) (100: 1 ratio combination)	randomised double-blind, placebo-controlled parallel	117 subjects with metabolic syndrome (aged 25–75 years)	8 weeks	↓ LDL-C, ↓non-HDL-C, ↓ TC, ↓ TG, ↓ Lp(a), ↑ HDL-C compared with the placebo	sdLDL	Panahi et al. 2014 [163]
1 g/day of curcuminoids (500 mg C3Complex® + 5 mg bioperine® Sami Labs LTD, Bangalore, Karnataka, India)	randomised, double-blind, placebo-controlled, crossover	30 obese subjects (mean BMI 33.95 ± 3.81)	30 days		BMI and weight compared with the placebo	Esmaily et al. 2015 [186]
50 mg/day of curcumin (250 mgCurcuWIN) or 200 mg/day of curcumin (1000 mg CurcuWIN)	randomised controlled double-blind, parallel	59 healthy subjects aged 19–29 years	8 weeks	200 mg/ day of curcumin: ↑ endothelial function compared with placebo		Oliver et al. 2016 [237]

↑—increased; ↓—decreased; ALT—alanine aminotransferase; AST—aspartate aminotransferase; BDNF—brain-derived neurotrophic factor; BMI—Body Mass Index; BUN—urea nitrogen; CHD—coronary heart disease; CRP—C reactive protein; EGF—epidermal growth factor; ET-1—endothelin-1; FBFACh—forearm blood flow to brachial artery infusion of acetylcholine; FFAs—free fatty acids; FMD—flow-mediated dilation; GPx—glutathione peroxidase; HbA1c—glycated haemoglobin; HDL- C—high-density lipoprotein cholesterol; HOMA-IR—homeostasis model assessment insulin resistance; LDL-C—low-density lipoprotein cholesterol; Lp(a)—lipoprotein a; LPL—lipoprotein lipase; LPS—lipopolysaccharides; MDA—Malondialdehyde; MCP-1—monocyte chemoattractant protein 1; NAFLD—non-alcoholic fatty liver disease; Nrf2—nuclear factor erythroid 2–related factor 2; NQO1—NAD(P)H: quinone oxidoreductase; OGTT—oral glucose tolerance test; PWV—pulse wave velocity; QUICKI—quantitative insulin sensitivity check index; SBP—systolic blood pressure; sdLDL—small dense low density lipoprotein; SOD—superoxide dismutase; TBARS—thiobarbituric acid reactive substances; TC—total cholesterol; TG—triglyceride; TGF-β—transforming growth factor beta; T2DM—type 2 diabetes mellitus; TNF-α—tumor necrosis factor-alpha; VLDL—very low-density lipoprotein; VEGF—vascular endothelial growth factor.
